# Spatial working memory in a disappearing object task is impaired in female but not male dogs with chronic osteoarthritis

**DOI:** 10.1007/s10071-024-01845-x

**Published:** 2024-03-02

**Authors:** Melissa Smith, Joanna C. Murrell, Michael Mendl

**Affiliations:** 1 Bristol Veterinary School, University of Bristol, Langford House, Langford, BS40 5DU UK; 2 Highcroft Veterinary Referrals, 615 Wells Rd, Whitchurch, Bristol, BS14 9BE UK

**Keywords:** Spatial working memory, Disappearing object task, Osteoarthritis, Chronic pain, Dog

## Abstract

Chronic pain in humans is associated with impaired working memory but it is not known whether this is the case in long-lived companion animals, such as dogs, who are especially vulnerable to developing age-related chronic pain conditions. Pain-related impairment of cognitive function could have detrimental effects on an animal’s ability to engage with its owners and environment or to respond to training or novel situations, which may in turn affect its quality of life. This study compared the performance of 20 dogs with chronic pain from osteoarthritis and 21 healthy control dogs in a disappearing object task of spatial working memory. Female neutered osteoarthritic dogs, but not male neutered osteoarthritic dogs, were found to have lower predicted probabilities of successfully performing the task compared to control dogs of the same sex. In addition, as memory retention interval in the task increased, osteoarthritic dogs showed a steeper decline in working memory performance than control dogs. This suggests that the effects of osteoarthritis, and potentially other pain-related conditions, on cognitive function are more clearly revealed in tasks that present a greater cognitive load. Our finding that chronic pain from osteoarthritis may be associated with impaired working memory in dogs parallels results from studies of human chronic pain disorders. That female dogs may be particularly prone to these effects warrants further investigation.

**Supplementary Information:**

The online version contains supplementary material available at 10.1007/s10071-024-01845-x.

## Introduction

In humans, chronic pain is often associated with cognitive deficits (Moriarty et al. 2011) including in facets of executive function such as working memory (Berryman et al. 2013) and spatial working memory (Antepohl et al. 2003; Luerding et al. 2008). There are several hypothesised mechanisms by which this association could arise (Moriarty et al. 2011). Chronic pain may increase the allocation of attention and working memory to pain-related processing, thus reducing the availability of these capacity-limited resources for other cognitive tasks (Apkarian et al. 2004a). Chronic pain is also associated with affective disorders (Leino and Magni 1993; McWilliams et al. 2003; Ohayon and Schatzberg 2003) and impaired sleep (Drewes et al. 2000; Nicassio and Wallston 1992; Riley et al. 2001), both of which have been shown to impair working memory (Christopher and MacDonald 2005; Harvey et al. 2004; Miyata et al. 2013; Rose and Ebmeier 2006; Steenari et al. 2003). Chronic pain can cause structural and functional changes in brain areas such as the medial prefrontal cortex, amygdala and posterior cingulate cortex (Baliki et al. 2008; Moriarty et al. 2011), all of which play roles in executive function and other cognitive and decision-making processes (Apkarian et al. 2004b). In contrast to research in humans, the extent to which chronic pain influences cognitive function in non-human animals has received relatively little study. Most work in this area has been done on laboratory rodents, mainly using models of induced pain (Mor et al. 2017; Moriarty et al. 2011; Negrete et al. 2017). However, an understanding of the relationship between spontaneous and naturally occurring chronic pain conditions and cognitive function in species kept and managed by humans is important from both a scientific and animal welfare perspective. For example, long-lived companion animals such as domestic dogs and cats are especially prone to the development of chronic pain conditions as they age. If these lead to cognitive deficits, for example in executive functions such as working memory, they could impair the animals’ ability to engage with their owners and environment or to respond to training or novel situations, and this in turn may affect their quality of life. Additionally, recognition of links between cognitive deficits and chronic pain conditions could assist with the diagnosis of these conditions, as well as preventing changes in cognitive function from being wrongly attributed to other disorders such as age-related cognitive decline. One common disorder of dogs likely to induce chronic pain is osteoarthritis which affects approximately 200,000 dogs in the UK (Anderson et al. 2018). Dogs with osteoarthritis show behavioural (Hielm-Björkman et al. 2003; Wiseman et al. 2001) and gait (Conzemius et al. 2003; Moreau et al. 2003) changes which can be reversed with nonsteroidal analgesia (Hielm-Björkman et al. 2009; Moreau et al. 2003) or surgical treatment (Conzemius et al. 2003), suggesting osteoarthritis causes pain in dogs, as it does in humans (Arendt-Nielsen et al. 2010). Furthermore, dogs with osteoarthritis also display lower mechanical and thermal nociceptive thresholds than healthy control dogs (Hunt et al. 2018; Knazovicky et al. 2016), suggesting hyperalgesia, as well as increased temporal summation and impaired descending noxious inhibitory control (Hunt et al. 2018). These physiological signs indicate central sensitisation (Woolf 2011) and imply that the chronic pain observed in dogs with osteoarthritis shares many of the physiological changes observed in human chronic pain (Arendt-Nielsen et al. 2010). Here, we investigate whether naturally occurring osteoarthritis in dogs owned by the public is associated with changes in working memory as is the case in humans (Antepohl et al. 2003; Berryman et al. 2013; Luerding et al. 2008). Specifically, we compare the spatial working memory of dogs with and without chronic osteoarthritis using the disappearing object task devised by Fiset et al. (2003), which is well suited for use in dogs owned by the public as it can be performed in a single session lasting only a few hours (Smith et al. 2021). If dogs, like humans, show working memory deficits in chronic pain disorders, this also increases the validity of using spontaneous canine osteoarthritis as a model for human chronic pain, thus offering a 3Rs Replacement and Reduction alternative to models of induced pain used in laboratory-housed animals (Guhad 2005).

## Methods

### Animals

Forty-one dogs were recruited via a social media campaign and poster and leaflet placement within veterinary clinics in Bristol and North Somerset. All recruited dogs had been neutered and 20 (12 females, 8 males) were assigned to the osteoarthritis group with a further 21 (6 females, 15 males) making up the control (non-osteoarthritis) group. In the absence of a similar study on working memory, and because this experiment was part of a wider project investigating effects of canine osteoarthritis on a range of readouts, we based our sample size on the work of Knazovicky et al. (2015) who used a crossover design (N = 19) to examine whether NSAID-treated and placebo-treated osteoarthritic dogs differed in measures of night-time sleep quality. Allocation to groups was based on a clinical examination using a standardised check list and administered by a veterinary surgeon (MS). In addition, MS asked owners about any signs of osteoarthritis, including stiffness, pain, slowing down during walks, difficulty jumping or climbing, and this also informed group allocation. Further details are provided in Smith et al. (2022), and information on the subjects is presented in Table 1.

**Table 1:** Signalments of participant dogs

Variable	Group	
	Control	Osteoarthritis
Sex		
Female (neutered)	6	12
Male (neutered)	15	8
Breed Class		
Gundog	10	9
Crossbred	4	3
Hound	1	0
Pastoral	1	4
Terrier	2	2
Utility	1	0
Working	2	2
Analgesia provision		
No	21	11
Yes	0	9
Analgesia frequency		
None	21	11
Occasional	0	3
Daily	0	6
Analgesia type		
None	21	11
Nonsteroidal analgesia only	0	6
Other analgesics provided	0	3
Season when dog participated		
Summer (May–August)	12	13
Winter (November–February)	9	7
Vet-assigned severity score		
None	21	0
Mild	0	11
Moderate	0	7
Severe	0	2
Owner-assigned severity score		
None	21	1
Mild	0	9
Moderate	0	9
Severe	0	1
Object used in task		
Tennis ball	11	8
Squeaky ring	10	10
Both objects used	0	1
Own toy	0	1
Continuous variables		
Age (years)	7.85 ± 0.50	8.10 ± 0.68
Body Condition Score	4.67 ± 0.42	5.45 ± 0.63
SNoRE Score	19.95 ± 3.93	20.42 ± 3.90
HCPI Score	4.14 ± 1.97	14.90 ± 3.31
CBPI Severity Score	0.0375 ± 0.0557	2.04 ± 0.974
CBPI Interference Score	0.0750 ± 0.1058	1.86 ± 0.774
CBPI Quality of Life (QOL) Score	3.70 ± 0.42	3.33 ± 0.32
CCDR Score	25.75 ± 0.74	27.30 ± 0.99

### Apparatus

**Fig. 1:**
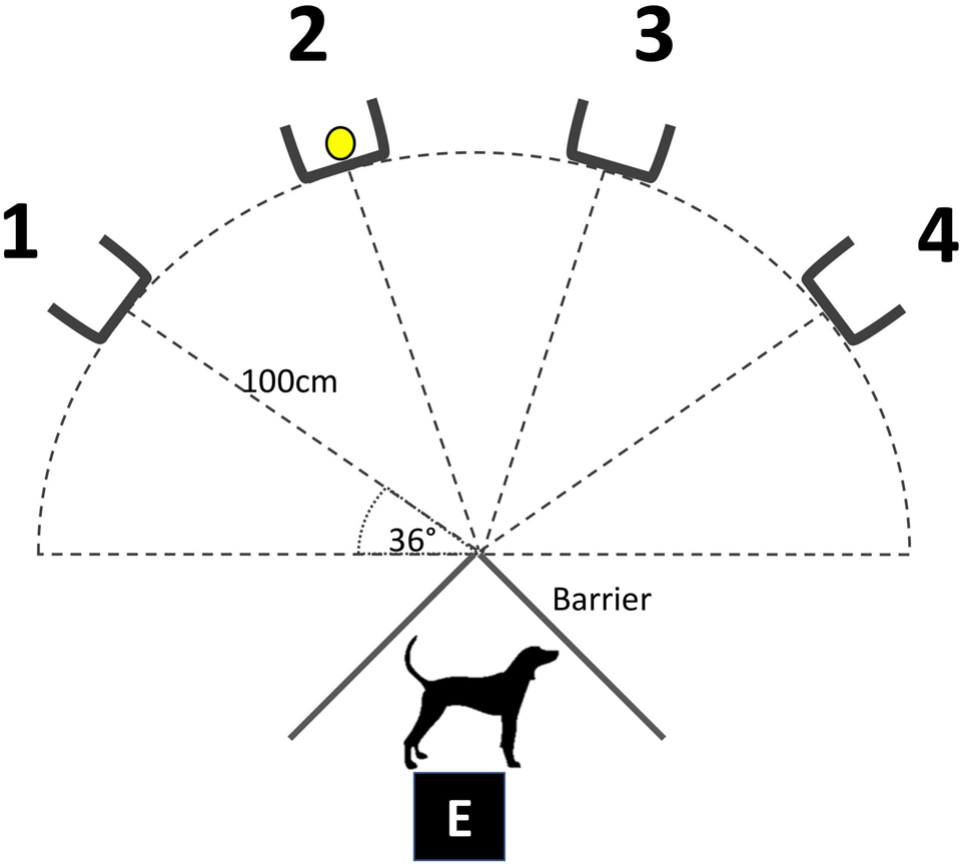
Diagram of the apparatus used in the disappearing object task. The position of boxes 1–4 relative to the dog’s starting position and position of the barrier during the memory retention interval are shown, with the object currently behind box 2. Distances and angles (measured using a tape measure and whiteboard protractor) are represented by black dotted lines. *E* represents the location of the two researchers during the retention interval

The disappearing object task apparatus comprised four identical cuboidal open-backed wooden boxes (30 cm high, 20 cm wide, 15 cm deep) each containing one of four identical carrier bags of 800 g aquarium gravel for weighting. A transportable barrier to prevent the dog seeing the boxes at certain times during the task was constructed from a folding two-panel laundry airer (each panel 102 cm high × 62 cm long) covered with opaque black plastic sheeting. These pieces of apparatus were arranged as shown in Fig. 1. The object to be hidden consisted of either a tennis ball or squeaky rubber ring toy attached to a 125 cm long, 1 mm thick transparent nylon thread. All equipment was cleaned between uses with F10 disinfectant spray (Health and Hygiene (Pty) ltd., Florida Hills, South Africa).

### Procedure

The task was performed in each owner’s own home by two experimenters, E1 and E2. A clinical examination including an orthopaedic examination of all appendicular joints was performed on each dog by a veterinary surgeon (E1: MS). A clinical history was also taken from each owner regarding any clinical signs of osteoarthritis or potential signs of other health problems. This information was used to assign each dog to the osteoarthritic or healthy control group. Both the dog’s owner and veterinary surgeon were independently asked to score the severity of the dog’s clinical signs of osteoarthritis as “none”, “mild”, “moderate” or “severe”. The disappearing object task apparatus was set up and the dog was then shown both object types (tennis ball and rubber ring). The object that was most preferred by the dog was selected for use, according to E1’s subjective opinion (based on initial object approached, relative duration of interaction with each object and presence of tail-wagging and jumping behaviours). One osteoarthritic dog showed no interest in either object and so their own preferred toy (a blue plastic ring) was used instead. The test procedure was adapted from that of Fiset et al. (2003; see also Smith et al. 2021) and consisted of three phases, shown in Table 2. In each trial, E2 held the dog by the collar whilst E1 placed the object in the required position (see Table 2) and returned to the position indicated in Fig. 1, before E2 released the dog. In the shaping phases, the dog received a reward (food, verbal praise and petting) when they touched the object with their nose, mouth or paw, and were then led back to the start point by E2 ready for the next trial. In the training and testing phases, the object was held by a string and moved in front of all boxes and then behind the target box (i.e. if the target box was box 3 or 4, the object was moved in front of boxes 1, 2, 3 and 4 and then behind the target box; if the target box was 1 or 2, the object was moved in front of boxes 4, 3, 2 and 1 and then behind the target box). The dog was able to observe the object being positioned and then the opaque barrier was placed in front of the dog (as shown in Fig. 1) for a specified memory retention interval (0 s during training; 0, 1 or 2 min during testing). The barrier was then removed and the dog was released with E1 looking towards the back of the test area (away from the boxes) and E2 looking towards E1 so as not to provide any visual cues as to the location of the object. For the 0 s interval, the barrier was put in place and then immediately removed, so the retention interval was likely c.2 s. However, for simplicity, we code it as 0 min in the data analysis (see below). If, following release, the dog visited (looked behind) the target (correct) box, they received a reward and were then led back to the starting position by E2 ready for the next trial. If the dog visited a box other than the target box or did not visit any box within 60 s of release, they received no reward and were led by E1 back to the starting position ready to begin the next trial. Dogs were not allowed to visit more than one box per trial. One dog showed visible signs of distress during the task and the task was immediately halted and the dog not included in the study. Apart from this, none of the osteoarthritic dogs displayed signs of distress or pain, and most dogs showed positive affective behaviours (e.g. tail wagging, jumping up, play bows, following the experimenter). The testing phase involved three trials for each box, one per memory retention interval of 0, 1 and 2 min (based on Smith et al. (2021)), in order to vary the difficulty of the task and assess whether and how the retention interval affected the success rates of osteoarthritic and control dogs. The target box and interval used on each trial was pseudorandomly generated using the RAND() function in Microsoft Excel, such that each combination of box and interval occurred once and the same target box or interval did not occur in two consecutive trials. On each trial, the first box visited was recorded and noted as either a ‘success’ (correct box) or ‘fail’ test outcome. During each home visit, owners were instructed to complete four questionnaires: the Helsinki Chronic Pain Index (HCPI) (Hielm-Björkman et al. 2009) and Canine Brief Pain Inventory (CBPI) (Brown et al. 2008) to assess the owner’s subjective opinion of their dog’s chronic pain; the Sleep and Night Time Restlessness Evaluation (SNoRE) to assess their dog’s sleep quality (Knazovicky et al. 2015) (as impaired sleep is common in human chronic pain disorders (Menefee et al. 2000) and may impair working memory (Chee and Choo 2004; Steenari et al. 2003)); the Canine Cognitive Dysfunction Rating Scale (CCDR) (Salvin et al. 2011) to screen dogs for clinical signs of age-related cognitive decline which could adversely affect task performance.

**Table 2:** Phases of the disappearing object task

Phase	Object location and barrier placement (if present)	Criterion for reaching next phase
Shaping Phase (Stage 1)	Object placed on floor next to dog, dog encouraged verbally and by pointing to touch the object (successful trial recorded when object is touched)	5 successful consecutive trials
Shaping Phase (Stage 2)	Object moved slightly closer to boxes with each successful trial	Object reaches boxes
Shaping Phase (Stage 3)	Object placed midway between two (randomly selected) boxes	10 successful trials
Shaping Phase (Stage 4)	Object placed behind box	8 trials (2 per box)
Training phase	Object placed behind box and barrier put in place momentarily (0 s to habituate dog to presence of barrier) then removed before releasing dog	12 trials (3 per box)
Testing phase	Object placed behind box and barrier then put in place for either 0, 60 or 120 s (memory retention interval) before releasing dog	12 trials (1 per box per retention interval)

### Data analysis

Data were analysed using mixed-effects logistic regression models in R using the lme4::glmer() function with the ‘binomial’ family specification. The success/failure of the dog on each trial during the testing phase was the binary outcome variable in all models. Given the sample size, we focused on the effects of group (osteoarthritis/control), sex (male/female), age and retention interval (0, 1, 2 min) as the key predictors of interest for our research questions, and included interactions between group and the latter three variables. As a sense check, we used univariate models to evaluate whether other signalment variables (Table 1) influence trial success/failure of dogs and hence might warrant inclusion in a final model (e.g. Alves et al. 2002; Bogaert et al. 2005; Kooby et al. 2003). Because the interaction of each variable with group (osteoarthritis/control) was considered of greater importance than the main effect of each variable, each univariate model contained both main and interaction effects. We, therefore, only ran these analyses on continuous variables, and on categorical variables with roughly balanced cell sizes at each level across the two groups (‘Season when dog participated’, ‘Object used in task’). The Quality of Life score on the CBPI was transposed to a number with “poor” given a score of 0 and “excellent” given a score of 4. ‘Dog id’ was included as a random effect. None of the univariate analyses revealed a significant effect of these other variables (see Supplementary Table S1), hence supporting our focus on the main predictors of interest in the analysis. To assess the significance (*p* < 0.05) of predictor variables and interactions in the final GLMM, we used likelihood ratio tests (LRT) to, non-sequentially, compare the full model with the model minus the predictor of interest. Mann–Whitney *U*-tests and Fisher’s exact tests were used to compare signalment variables between groups.

## Results

**Fig. 2:**
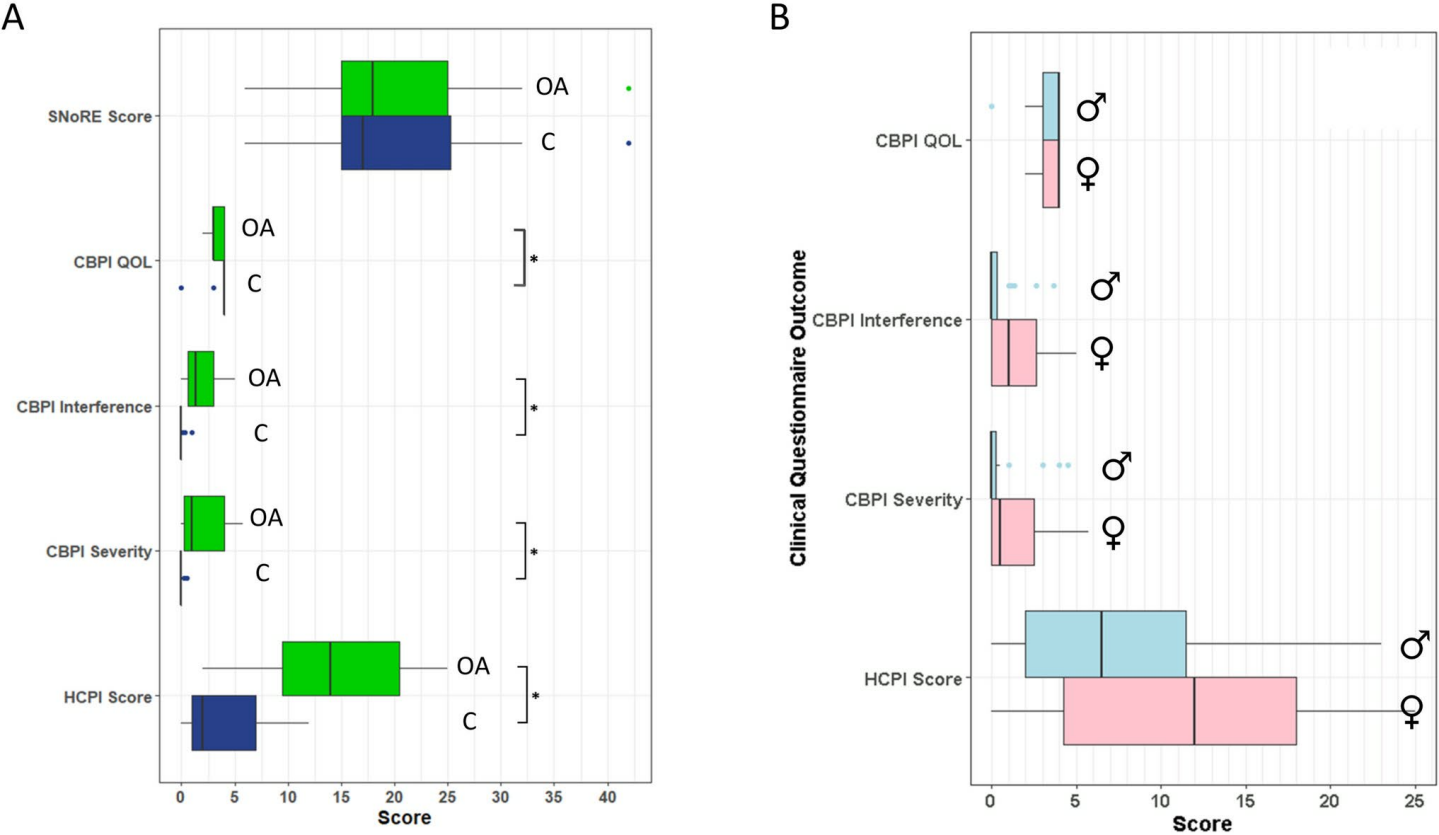
The effects of (**A**) group on SNoRE, CBPI and HCPI questionnaire scores (OA = osteoarthritis group; *C* = control group), and (**B**) sex on CBPI and HCPI questionnaire scores (♂ = male; ♀ = female). Asterisks denote significant differences (*p* < 0.05) between groups

The signalments of dogs recruited in each group are shown in Table 1. Analysis of continuous variables showed that osteoarthritic dogs had significantly higher HCPI (*U* = 39, *p* = 1.39 × 10^–5^), CBPI Severity (*U* = 56, *p* = 2.57 × 10^–5^), and CBPI Interference (*U* = 49.5, *p* = 1.84 × 10^–5^) scores, and lower CBPI Quality of life (*U* = 250, *p* = 0.01497) scores than control dogs, but the two groups did not differ in SNoRE score (*U* = 181, *p* = 0.8109; Fig. 2A). We investigated whether osteoarthritis was more severe in females than males (as is the case in humans: Affleck et al. 1999; Keefe et al. 2000; Srikanth et al. 2005) but whilst median scores for all clinical questionnaire components except for the CBPI QOL score were higher in female osteoarthritic dogs, this effect was not significant (Holm-Bonferroni-adjusted α-thresholds) for any questionnaire score (Fig. 2B) nor was there any significant difference between the vet-assigned severity scores (Fisher’s exact test, *p* = 0.796) or owner-assigned severity scores (Fisher’s exact test, *p* = 0.749) of male and female osteoarthritic dogs. There were no significant differences between groups in age (*U* = 186, *p* = 0.5284), sex (Fisher’s exact test, *p* = 0.0616) or body condition score (*U* = 140.5, *p* = 0.0554). No dogs had a CCDR score of 50 or more (the threshold for probable cognitive dysfunction (Salvin et al. 2011).

**Fig. 3:**
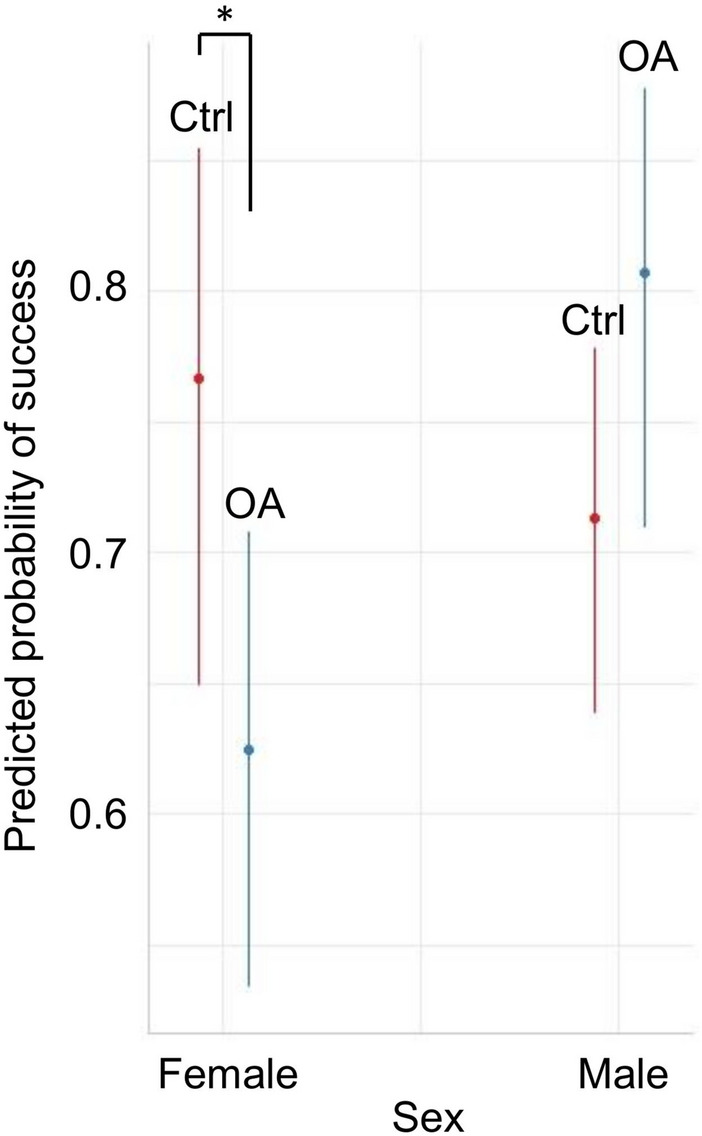
The effect of group and sex on predicted probability of success during the disappearing object task. Asterisks denote significant differences (*p* < 0.05) between osteoarthritic (OA) and control (Ctrl) dogs of the same sex

There were no main effects of Group, Sex or Age on success/failure in the task (Group: Beta = − 1.98, LRT = 0.365, *p* = 0.546; Sex: Beta = − 0.278, LRT = 1.73, *p* = 0.188; Age: Beta = − 0.244, LRT = 1.40, *p* = 0.236). However, there was a significant Group by Sex interaction (Beta = 1.198, LRT = 6.27, *p* = 0.012). Post hoc testing indicated that this was principally due to female (*z* = 1.964, *p* = 0.049), but not male (*z* = − 1.574, *p* = 0.115) osteoarthritic dogs showing lower proportions of successful visits than control dogs of the same sex (Fig. 3). No significant Group x Age interaction was detected (Beta = 0.239, LRT = 1.87, *P* = 0.171).

**Fig. 4:**
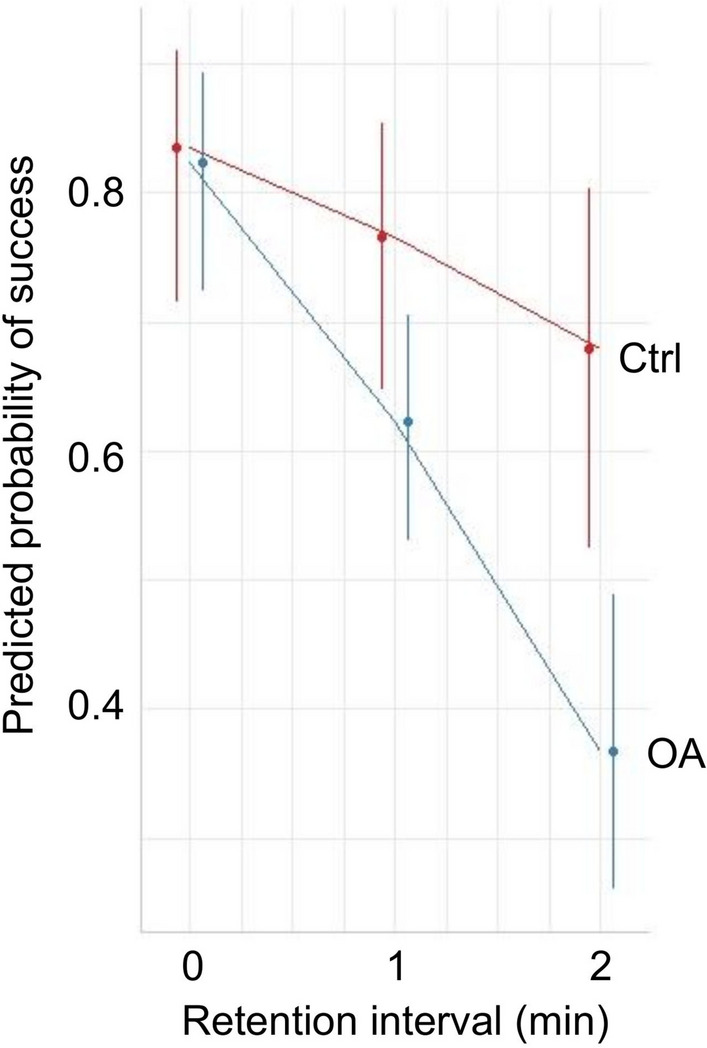
The effect of memory retention interval on predicted probability of success for osteoarthritic (OA) and control (Ctrl) dogs

There was a significant effect of memory Retention Interval (Beta = − 0.434, LRT = 32.32, *p* < 0.001). Predicted probability of success decreased with increasing interval duration as expected (Fig. 4). However, there was also a significant Group x Retention Interval interaction (Beta = − 0.611, LRT = 5.21, *P* = 0.022), with osteoarthritic dogs showing a steeper decline in performance with increasing retention interval than control dogs (Fig. 4).

## Discussion

Dogs were assigned to the osteoarthritic and control groups on the basis of clinical examination and clinical history. Differences in the Helsinki Chronic Pain Index (HCPI) (Hielm-Björkman et al. 2009) and Canine Brief Pain Inventory (CBPI) (Brown et al. 2008) scores indicate that dogs in the osteoarthritis group were indeed experiencing more severe pain, functional impairment and decreased quality of life compared to control dogs, hence supporting the assignments made. Analysis of the disappearing object task indicated that female osteoarthritic dogs made fewer successful visits to the target boxes than female control dogs, whilst the same relationship was not observed for male dogs. Thus, female but not male neutered osteoarthritic dogs exhibited impaired spatial memory in the disappearing object task compared to control dogs of the same sex and neutering status. It seems unlikely that this effect was mediated by the interplay between sex-specific hormones (cf. Frye et al. 2004; Gouchie and Kimura 1991) and osteoarthritis (or the pain it induced), because all dogs in the study were neutered. However, osteoarthritis is more common and severe in human women than men (Affleck et al. 1999; Keefe et al. 2000; Srikanth et al. 2005), especially following the menopause (Lawrence et al. 1966; Silman and Newman 1996; Wluka et al. 2000), with ovariectomised rats (Hoegh-Andersen et al. 2004) and sheep (Cake et al. 2005) being used in previous studies to model this effect. Therefore, it is possible that ovariectomised female dogs in this study may also have had more severe osteoarthritis than male dogs. However, whilst the median HCPI and CBPI Interference scores were slightly higher for female than male osteoarthritic dogs, this difference was not statistically significant, implying that female osteoarthritic dogs did not have markedly more severe osteoarthritis than male dogs in this study.

Female mammals often perform less well in spatial memory tasks than male mammals (Jones et al. 2003; Jones and Healy 2006), so it is possible that the combination of this effect and spatial working memory impairment from (the pain of) osteoarthritis was sufficient to cause a significant decrease in performance in osteoarthritic female dogs, but neither factor alone was sufficient to cause decreased performance in healthy female dogs or male osteoarthritic dogs. A related finding was reported by Harris et al. (2008b) who found that acute stress (as may co-occur with a pain state) resulted in behavioural changes and poorer performance in a spatial memory task in female but not male adult rats, although there was no evidence that chronic stress, as may be induced by longer-term osteoarthritic pain, had a similar effect (Harris et al. 2008a, 2008b).

The decrease in probability of success with increasing memory retention interval is similar to that observed by Fiset et al. (2003) and Smith et al. (2021) and suggests that dogs were indeed using working memory to locate the object rather than relying on cues such as scent, in which case they would likely have shown similar success rates at all intervals. Interestingly, osteoarthritic dogs showed a steeper decline in performance with increasing retention interval compared to control dogs, suggesting that effects of the condition on cognitive function were more clearly revealed as the task became more difficult. There is some evidence for a similar interaction between cognitive load and the impact of chronic pain in humans, although most data come from acute pain models, and there are conflicting findings (e.g. Moore et al. 2017). Recent research on rats has also demonstrated that detrimental effects of chronic pain on memory performance are more clearly observed in more demanding tests. In a novel object recognition task, subjects experiencing pain performed more poorly when exposed to difficult (similar objects) as compared to easier (very different objects) versions of the task across both short and longer term retention intervals. In contrast, control animals performed equally well under both easy and difficult conditions (Phelps et al. 2021). The authors proposed that chronic pain can occupy cognitive resources such that insufficient cognitive capacity is available to solve more difficult tasks. A similar explanation may apply to our current findings and, at the very least, they emphasise that varying task complexity can allow us to glean useful information about the interaction between cognitive load and the effects of pain on task performance, and to reveal differences that may go unnoticed if only easy versions of tasks are used.

In summary, our study illustrates the potential for field investigations of cognitive abilities, including working memory components of executive function, in dogs owned by the public. Such in-home investigations are likely to be less stressful for dogs than those that require them to be tested in an unfamiliar laboratory setting, and hence will provide data that is relatively uncontaminated by incidental effects of acute stress on cognitive function (Harris et al. 2008b; Mendl 1999). They can also start to reveal the effects of spontaneous changes in health on cognitive ability in this key companion animal species, which are important in their own right from an animal welfare perspective and may also be of use in helping to understand and model human health and ageing (Hoffman et al. 2018). To this end, our study is the first to indicate that some dogs with osteoarthritis and associated chronic pain may have working memory deficits, a finding which has clear parallels in human chronic pain disorders (Berryman et al. 2013). That these may be particularly pronounced in females is of interest and warrants further investigation.

## Supplementary Information

Below is the link to the electronic supplementary material.

Supplementary file1 (DOCX 28 KB)

## Data Availability

Data are at: https://figshare.com/s/d735b73229158c2f7487.
