# Supplementary material for: Spatial working memory in a disappearing object task is impaired in female but not male dogs with chronic osteoarthritis
Source: Anim Cogn. 2024 Mar 2;27(1):13. doi: 10.1007/s10071-024-01845-x (PMC10907419; doi:10.1007/s10071-024-01845-x)
Supplement: Supplementary file 1 — Supplementary file1 (DOCX 28 KB) [file 10071_2024_1845_MOESM1_ESM.docx]

**Supplementary Material**

**Spatial working memory in a disappearing object task is impaired in female but not male dogs with chronic osteoarthritis**

Melissa Smith, Joanna C. Murrell^1^, Michael Mendl*^2^

*Bristol Veterinary School, University of Bristol, Langford House, Langford, BS40 5DU, UK*

^1^ Present address: Highcroft Veterinary Referrals, 615 Wells Rd, Whitchurch, Bristol, BS14 9BE, UK

* Corresponding author: [mike.mendl@bris.ac.uk](mailto:mike.mendl@bris.ac.uk)

^2^ ORCID: 0000-0002-5302-1871

**Table S1** Results of the univariate models.

|  | **Main effect** | | | | | **Interaction with Group** | | | | |
| --- | --- | --- | --- | --- | --- | --- | --- | --- | --- | --- |
|  |  | **95% confidence limits** | |  |  |  | **95% confidence limits** | |  |  |
| **Categorical variables** | **Odds Ratio** | **Lower** | **Upper** | **z-value** | **p-value** | **Odds Ratio** | **Lower** | **Upper** | **z-value** | **p-value** |
| **Object used in task** |  |  |  |  |  |  |  |  |  |  |
| Tennis ball | Ref. | - | - | - | - | Ref. | - | - | - | - |
| Squeaky ring | 0.685 | 0.371 | 1.267 | -1.206 | 0.228 | 1.259 | 0.520 | 3.050 | 0.51 | 0.61 |
| Own toy | 0.873 | 0.214 | 3.558 | -0.19 | 0.849 | - | - | - | - | - |
| Both | 0.652 | 0.151 | 2.814 | -0.573 | 0.566 | - | - | - | - | - |
| **Season when dog participated** |  |  |  |  |  |  |  |  |  |  |
| Summer (May-August) | Ref. | - | - | - | - | Ref. | - | - | - | - |
| Winter (November-February) | 0.715 | 0.385 | 1.327 | -1.063 | 0.288 | 1.678 | 0.691 | 4.072 | 1.144 | 0.253 |
| **Continuous variables** |  |  |  |  |  |  |  |  |  |  |
| Body Condition Score | 1.130 | 0.799 | 1.598 | 0.693 | 0.489 | 0.755 | 0.500 | 1.140 | -1.337 | 0.181 |
| SNoRE Score | 0.989 | 0.952 | 1.027 | -0.589 | 0.55568 | 1.010 | 0.957 | 1.066 | 0.360 | 0.71879 |
| HCPI Score | 1.046 | 0.970 | 1.128 | 1.164 | 0.24453 | 0.935 | 0.856 | 1.020 | -1.510 | 0.13111 |
| CBPI Severity Score | 2.252 | 0.108 | 47.146 | 0.523 | 0.601 | 0.471 | 0.022 | 9.897 | -0.485 | 0.628 |
| CBPI Interference Score | 2.659 | 0.429 | 16.480 | 1.051 | 0.293 | 0.351 | 0.056 | 2.201 | -1.117 | 0.264 |
| CBPI Quality of Life (QOL) Score | 0.709 | 0.427 | 1.179 | -1.324 | 0.1855 | 1.101 | 0.544 | 2.228 | 0.269 | 0.7881 |
